# Intelligence, &c.

**Published:** 1827-10

**Authors:** 


### INTELLIGENCE, CORRESPONDENCE, &c.

### OBITUARY—MR. SHAW.

Pallida mors æquo pede pulsat pauperum tabernas

Regumque turres.

Vitæ summa brevis spem nos vetat incho are longam.

Jam te premet nox, fabulæque manes,

Et domus exilis Plutonia.

It is our painful duty this quarter to record the decease of Mr. Shaw, which took place on the 19th July, in the 36th year of his age. This excellent anatomist, and deserving young surgeon was attacked with a formidable and protracted fever towards the end of May; but, after a time, he became so far convalescent as to bear removal into the country, and the most sanguine expectations were entertained of his ultimate recovery. On the 17th of July last, however, he experienced a sudden relapse, and survived only a few days.

Mr. Shaw was, for many years, Demonstrator of Anatomy in the School of Great Windmill Street, and took a very active part in the formation of the Anatomical and Pathological Museum of Mr. Charles Bell, a collection, we believe, almost unrivalled for the beautiful manner in which the preparations are put up. On the death of Mr. Wilson, Mr. Shaw became co-lecturer with Mr. Bell, and not long ago he succeeded Mr. Cartwright as surgeon to the Middlesex Hospital.

That Mr. Shaw was no drone, is evident from his writings. The Manual of

Anatomy was happily conceived and not less happily executed. Whilst it laid before the student the details and minutiæ of muscles, and ligaments, and so forth, it directed his attention to the pathological conditions, which those structures are apt to take on, and we do not hesitate to assert has been, and will be, of very essential service to the Junior Members of the Profession. His works upon the Spine evince considerable ingenuity and originality; and though, perhaps, some of his peculiar views may be carried a little too far, there is not a doubt that they are founded in truth and correct observation. Besides these larger works, his numerous papers on the nerves and other subjects, in our different Medical Journals, are strong proofs of able industry.

As a man, and particularly as a teacher, Mr. Shaw will be universally regretted. With the pupils of Windmill Street, he was popular to a degree, for his manners were of that frank, independent character which always tells upon the hearts of young men. He was not, as some are, reserved and supercilious, but entered into the feelings and sympathies of his pupils, and was in word and in deed one of them. Whether his labours in the cultivation of anatomy, may have impaired an originally robust constitution we know not; but certainly, looking at Mr. Shaw, no one could have supposed it likely that he would have been cut off thus early. It is a melancholy circumstance when we see the youngest and the strongest swept away in their prime, whilst grey-beards still cling convulsively to a scarcely enviable existence; but it is still more melancholy to think, that men, like Mr. Shaw, after years of toil, should not be allowed to gather what they had sown, but should be called to their account in another world, just as they were beginning to taste the sweets of this!

To Mr. Bell, the loss will be great, but the family and friends of the departed, may console themselves with the reflexion that he died with a fair fame and unspotted reputation. From our hearts we say,

"Peace to his manes!"

---

*Artificial Anatomical Preparations.*

The professional and many of the non-professional gentlemen of London have been lately much gratified by an inspection of Dr. Ameline's Artificial Anatomical preparations in Golden Square. These preparations exhibit a striking example of the extent to which the ingenuity of an individual will reach, when under the guidance of persevering and patient industry. It is impossible to convey any adequate conception of these models, without the aid of the eye. They are not so beautiful as models in wax, no doubt; but they have the great advantage of bearing to be handled and pulled asunder without any detriment. The human fabric opens like a clock, and unfolds all its intricate apparatus of the natural size and natural appearance. The viscera all take out, and thus shew us all the interior machinery, one wheel after another. We understand M. Ameline asks 4000 guineas for these models. If they could be procured at a more moderate price, they would afford an excellent illustration of a *popular* course of Lectures on Anatomy and Physiology. In a professional point of view, nothing, of course, can prove a substitute for the living and the dead body. It is on these alone that the anatomy of the human fabric, and its various derangements can be studied.

---

*Dr. Hopkins' Obstetric Machine.*

In connexion with M. Ameline's models, we may mention in this place a most ingenious apparatus invented and constructed by Dr. Hopkins, of Westminster, whereby the process of human parturition is more closely imitated, than by any other means which we have ever witnessed. The fœtus, as well as the parts through which it passes, are constructed of elastic gum, and the natural evolution of the fœtus and placenta is closely and admirably imitated. It is needless to say that, by means of such a machine, the artificial aids of midwifery can be proportionally illustrated. The apparatus must be of essential benefit to the pupils of the ingenious Doctor's class.

---

*Dublin Hospital Reports.*

We are sorry the Editors of the 4th volume of the above Reports should have deemed it necessary to offer any explanation of a circumstance adverted to at page 112 of our last Number. We were perfectly aware of the *manner* in which the apparent *favouritism* was produced; and we never, for a moment, suspected that the respectable Editors were concerned in the matter. The affair is a mere bagatelle, and we beg the Editors to accept, as the Diplomatists say, "the assurance of our high consideration."

*The Contented Helot, or Man in Mask.*

In the last Number of the Medical and Physical Journal, there is a Letter signed "A LICENTIATE of the LONDON COLLEGE of PHYSICIANS," but who is, unquestionably, some FELLOW in disguise.

We cannot but envy the happiness of this Helot's paradise. Every thing with this *soi-disant* Licentiate is in the most flourishing and delectable condition. The LICENCE gains him great respectability—admits him to be candidate (but never a successful one) for Hospitals and Dispensaries—in short, it is quite the summum bonum of every respectable Physician's aspirations; and there is not a malcontent in London or in England, except Dr. Harrison! He very kindly tells the College, therefore, that it is not worth their while to go to law with this "vaunting individual," when there is not another in London who shares his sentiments! There is one thing which comes out from this disinterested Physician—namely, an *idea* that the College will not gain much whichever way the verdict goes—and, in this, we entirely agree with the Man in Mask. With much NAIVETÉ he says—"where is the *equity* of the power thus vested in the College?"—to wit: of punishing the Graduate of Edinburgh for practising at Fulham, while at Richmond he might prescribe *ad libitum!* On another point we entirely agree with the Man behind the curtain, namely that "*the real and best sources of their (the College) power, is the good opinion of the public, which may be shaken, but cannot be increased by a public trial.*" In our next Number we shall bring forward some curious documents to shew what the general sentiments of the Profession are and have been on the present distinction between Fellows and Licentiates—some of them, documents signed by one of the most distinguished of the LIVING FELLOWS of the College itself. Till then, vive valeque, thou happy HELOT!

*Anatomical Persecution.*

In our last Number, we noticed the persecution (a more proper term than prosecution) of Mr. Cooke, of Exeter, and informed our readers that many of the very first professional characters in this metropolis had privately subscribed a sum of money to defray the legal expenses incurred by Mr. Cooke. We did not approve of this *mode* of subscribing; but circumstances have come to our knowledge which induce us to think it is, *for the present*, politic not to come forward publicly on the above-mentioned occasion, while a negociation is going on in a certain quarter, with the view of remedying the evil of which anatomists have so much reason to complain. Those few who have wished their names and subscriptions to appear publicly, will see them in another place, to which we have transmitted them.

This notice will satisfy Mr. Cooke that his persecution is not overlooked, though it cannot, at present, be publicly canvassed, for the reasons alluded to above.

*Omission of Cancel.*

In the 8th Number of this Series, page 588, there is a passage, in the report of the proceedings at the Freemason's Tavern, which Mr. Wakley considers as alluding to, and reflecting on himself personally—namely, as "one who is a disgrace to the Medical Profession, and who is banished from the practice of it." Wishing to avoid all interference with private character, and to separate it entirely from public writings or opinions, we can have no hesitation in declaring that we know nothing of Mr. Wakley personally, nor of any circumstances which can justify the above allegations as applied to him individually—and that we believe the said allegations to be without foundation. The passage was not cancelled at the time of the trial, in June 1826, because it was not included in the plaintiff's declaration—and the continuance of the passage in the cancelled Numbers of the Journal afterwards, was entirely without design, and indeed without the consciousness of the Editor, till his attention was drawn to it by the notice that new legal proceedings were about to be instituted on that account.

*Hospital Reports.*

Six important Hospital Reports reached us during the last quarter, and a seventh was nearly prepared for us, when *two of them* were interrupted (after one had actually been printed) by the seniors of certain hospitals, (which, *at present*, shall be nameless) on the plea that such reports are not *warrantable*—and, moreover, that the cases occurring in hospitals are the *private property* of the physicians and surgeons of such institutions, and, consequently, that none else have any right to publish them! We shall not condescend to argue with these petty tyrants, who would monopolize—not the *improvements* of medical science, but the whole of the ignorance and obstinacy of the healing art, to themselves. They dare not allow records of practice to see the light, unless *manufactured* by themselves! Their objections to anonymous hospital reports, where party-spirit and personalities are sometimes indulged, would have some feasible basis to rest on; but when a gentleman, who daily walks the hospital, offers to the world a faithful transcript of the facts presented to his view, authenticated by his own name, the objections to such publication must arise from causes which we dare not trust our feelings, at present, in characterising by their proper names. The personages in question, however, are on the eve of annihilation; and even the short span of their worthless existence which is yet to run, will assuredly be embittered by the complete frustration of their feeble, selfish, and unchristian endeavours to cramp the diffusion of useful information, lest their own imbecility should be exposed. The eagle eyes of the press are already gazing on the transactions of public institutions—and the present generation of students *will* bring before the public the good and the evil practice of hospitals—the former for imitation, the latter for reprobation. In doing so, they are violating no compact, so long as they keep to a simple record of facts, without partiality or misrepresentation. On the contrary, they are contributing to the erection of the most splendid edifice that ever reared its head in medical science. Let them not be intimidated by the threats of a few individuals, whose utter and reckless disregard for all public utility, for all honourable distinction—in short, for all things but selfish, paltry, pitiable pelf, is a disgrace to the philosophic spirit of the age in which we live. Students who choose to publish fair, accurate, and authenticated accounts of hospital practice, are under no obligation to consult the opinion, or solicit the sanction of MOLES, who hate the light—of INCUBI, who press, with all their leaden Lethean weight, on the vitals of the profession—of UPAS trees, in the field of science, whose presence marks the centre of a circle of sterility—of VAMPYRES, who would suck the stream of knowledge from public charities, and bury it in the earth, calling it *their* private property—in fine, of a few remnants of ignorance and VANDALISM, whom the temper of the TIMES, and the spirit of the PRESS, will soon scourge off the stage! In despite of these personages, the department of PRIZE HOSPITAL REPORTS in this Journal flourishes—and will continue to flourish. We have awarded two prizes this quarter, and shall award two or more prizes in our next.

And here we cannot help taking some credit to ourselves for thus giving such a decided impulse to the important act of recording and publishing Authenticated Hospital Reports. We have no hesitation in prognosticating that, if the practice becomes general, of which there is every appearance, it will be the most beneficially operative improvement that ever took place, in this or in any other country. The salutary effects will be felt in a variety of ways. The practice of taking accurate notes of hospital cases necessarily produces a habit of accurate observation, which will tell advantageously on every subsequent stage of the individual's professional life. The prize accorded to the student in this way, is the premium best adapted for his situation and years. His *perceptions* are all in the highest degree of acuteness, and it is the exercise of *these* which we want in the *faithful record of facts*. His judgment and reflections may be exercised to advantage at a later period. These hospital reports, guaranteed by the name of the reporter, prove at once a stimulus, and a check on the superior medical officers, while there is a perfect security against wilful misrepresentation on the part of the recorder. Thus the best effects will result from this system, as regards the medical officers, the students, and the institutions themselves. Of what inestimable advantage to the public will be these authentic records of hospital practice, we need not say. The most plodding routinist will not hesitate to consult these portraits of diseases in all their varied forms, for the sake of benefiting in his own private practice—while the most determined sceptic will not venture to hint any doubt of the *truth* of reports thus verified by the best of all vouchers—an appeal to the evidence of the senses of the whole of the officers and students of the hospital. The importance and utility of this system will soon be so manifest, that, ere long, a whole journal will probably be dedicated to BRITISH HOSPITAL REPORTS alone. Till that period arrives, we request the candidates for prizes in this Journal, to observe carefully, and record faithfully—to study brevity and perspicuity in their language—to curtail, as much as possible, the minute diurnal details, but preserve the prominent phenomena of the disease, the principal remedies employed, and the ultimate issue of events. By so doing, they will give an early proof of their assiduity to their friends—while they will confer an immense advantage on the profession and the public at large.

---

*The Chlorides of Soda and Lime.*

Chlorine, under the name of Oxymuriatic Acid, has been long known to possess active medicinal properties, and was applied by Mr. CRUIKSHANK and Dr. CRAWFORD in gangrene; by Dr. SCOTT, of Bombay, as a bath for liver complaints; and by Dr. BROWN, in atonic and syphilitic disorders; by GUYTON MORVEAU and others, as a fumigation, for the prevention of infection; extensively, by Mr. FARADAY, at the Penitentiary. From the inconvenience attending its production, the unmanageable and irritating nature of the uncombined gas, its use was necessarily confined to houses and apartments from which the inmates were previously removed, and its value as a medicine very much limited. For the purposes of bleaching, these difficulties had been long since overcome, by uniting it with Lime, which renders it capable of general application. M. LABARRAQUE was the first to point out the advantages to be derived from its application to medical purposes, in combination both with Lime and Soda; but, even in his preparations, the formulæ for which have been received into the French Materia Medica, the gas is too rapidly evolved, liable to excite irritation, and, by decomposing the water, to lose its peculiar properties.

The Chloride of Soda having been extensively and beneficially introduced into medical practice, it became a matter of the utmost importance, to obtain it of uniform strength and in perfect combination, upon both of which its valuable properties mainly depend. These desiderata have been obtained in the English preparation, which is three times the strength of that of the French Formula, and the evolution of the Chlorine is so gradual, as to be almost imperceptible to the most delicate patient, and, by excluding the air, it will keep for any length of time.

The Chlorides of Soda and Lime, besides being the most powerful antiseptics, possess the property of destroying all fetid exhalations, arising from animal and vegetable decomposition, by combining with the hydrogen, which is invariably one of the products resulting from such decomposition; and, to use the words of Mr. BRANDE, "as the effluvia which arise from putrescent substances, and more especially those generated in certain putrid disorders, have a tendency to create peculiar diseases, or to give the living body a tendency to produce poisons analogous to themselves;" the Chlorides destroy these, by acting chemically upon the pernicious matter, and resolving it into innocuous principles; and either of them may be efficaciously used to destroy the germ of all infectious diseases, by sprinkling the liquid, diluted with forty parts of water, about the chambers of the sick, suspending the use when its smell becomes perceptible; by immersing in it the linen, &c. of the patients, which should be rinced in pure water before being sent to the wash; and by receiving the discharges from the patient in vessels in which a wine-glass full has been previously added to the water.

The Chloride of Soda is preferable for medicinal purposes, as, at the same time that the Chlorine destroys the fetor of wounds, the Soda acts as a mild alkaline solvent of the proud flesh, and disposes the diseased parts to a healthy granulation. It has been found to act very beneficially, as a *Lotion*, in carbuncle, ill-conditioned ulcers and gangrenous sores of every description; fetid discharges of cancer, herpes ulcerans, porrigo favosa, atonic ulcers, ulcers of the uterus, mortification, tinea capitis, burns, scalds, &c.; as a *Gargle*, in ulcerated sore throats, ptyalism, spongy gums, and carious teeth; as an *Affusion*, in the hot stage of fevers, while the skin is dry; and in all infectious diseases; and as a *Stimulant*, in asphyxia and syncope. The *Proportions* to be used are best left to the judgment of the Medical Practitioner, and are varied with the circumstances of the case, from one in five to one in forty parts of pure water. No danger will arise from using the stronger proportions, as the Chlorine, from being so intimately combined, will be evolved much slower than when more diluted; it is probable, however, that frequent repetitions of the weaker will prove the most efficacious. As an *Internal Medicine*, it has been hitherto little used; but, as oxymuriatic acid has been given in scarlet and other fevers, in doses of eight to ten drops, and when it is considered that calomel and corrosive sublimate are chlorides of mercury, it is deserving of attention, whether Chlorine, in its milder combination with Soda, may not be beneficially substituted in many cases. Should the Chlorine be too rapidly evolved by the presence of an acid in the stomach, a solution of ammonia will give instant relief.

The Chloride of Lime, from its very moderate price, is best adapted for the general purposes of correcting, by occasional sprinkling, the confined air of workhouses, prisons, ships, courts of justice, and places of public resort; of purifying drains, sewers, cesspools, &c.; and of preserving the health of persons engaged in the deleterious processes of preparing oil colours, and smelting the ores of copper, lead, &c. It will be found essentially to promote health and comfort in hospitals, if, with the usual attention to fresh air and cleanliness, the diluted liquid be sprinkled twice a day about the wards: in this and similar instances, it acts by double affinity,—the Chlorine combining with the animal effluvia, and the Lime with the carbonic acid of exhaled air. For the purpose of fumigation, (the patients being removed,) it is only necessary to add a few ounces of dilute sulphuric acid to the requisite quantity of the pure liquid. FREDERIC FINCHAM.

*Manchester.*

*Factitious Mineral Waters prepared at the German Spa, Brighton.*

From the complicated nature of Mineral Waters, their chemical analysis would be found a very inadequate basis for a theory of their medical effects; a thorough acquaintance with which must be the result of observation alone. With respect to the Springs specified in the tables of analysis, as their peculiar character has been established by experience of long standing, it would be proper to give a general statement of the forms of disease wherein they prove salutary. The primary cause of a disease, and the progress it has made, are however considerations of equal moment, and must be referred to the judgment of the physician.

The present imitations are admitted, both by the faculty and the public of Germany, to coincide perfectly with the original Springs, as well in their modus operandi, as in their external properties. Mr. Faraday, of the Royal Institution, has analysed the artificial Carlsbad, one of the most complicated of the waters, and has allowed a reference to be made to him as to its chemical correctness.

*Note.*—We have particularly examined the apparatus employed in the formation of these artificial Mineral Waters, and we have no hesitation in saying that it is above all praise. We have had the testimony of some talented physicians at Brighton, touching the efficacy of these waters in various chronic disorders of the viscera, and it is most satisfactory. We have also seen many patients who had derived the greatest advantage from their use. They are highly deserving of the patronage of the profession generally, on account of their own intrinsic good properties, and the superior manner in which they are elaborated at an enormous expence. In our next Number, we shall give some further details of these important auxiliaries to medicine.—*Ed.*

---

MR. FROST'S BOTANICAL LECTURES.

We observe that Mr. Frost commences his Botanical Lectures at St. Thomas's Hospital, on the 1st of October 1827, and continues them every Wednesday, at half-past twelve afterwards. Mr. F. divides his course into three parts—*first*, contains the History of Botany—*second*, the arrangement of plants—*third*, Medical Botany. We have no doubt that Botanical Lectures will now be more numerously attended than ever, seeing how general the study is become among all classes of society. We wish Mr. Frost every success which his zeal and talents so well deserve.

*Medical Jurisprudence.*—We are glad to see that Dr. Gordon Smith, the well-known author of the "*Principles of Forensic Medicine,*" recommences his Course of Lectures on the above important subject, at the Royal Institution, on the first of October, at nine o'clock in the morning, and continues them at the same hour on Mondays, Wednesdays, and Fridays. From the prospectus which we have seen, and from the talents and acquirements of the Lecturer, we have no doubt that the Course will prove highly interesting, and ought to be carefully attended by all medical students in the western schools of the metropolis.

We learn that Dr. Burrows' long-expected work on Insanity is in the press, and will be published before Christmas.

*Midwifery.*

We have hitherto taken no notice of the strange *newspaper* publication of Sir A. Carlisle, because, to seriously controvert his positions before a *professional* tribunal, would be like a laboured attempt to prove that the sartorius muscle is longer than the pectoralis, or that bone is generally firmer in texture than flesh. Had the letter in question appeared without a signature, we should have concluded that it was indited in an asylum, by some incarcerated member of the medical profession; since, in a state of hallucination, the judgment, and even the passions, are often perverted. Thus, we hate those whom we loved before; we discredit realities; and we firmly believe in the most wild creations of a disordered imagination. But the author's name is a sufficient guarantee that the said letters are not the offspring of mental alienation, in the common sense of the word. The motives of men's actions lie deep in the recesses of the human breast, and God forbid that we should attempt to drag them from their dread abode. We are bound to give every man credit for sincerity and good intentions, while we submit his words and actions to the ordeal of inquiry, whatever might have been the motives impelling to these words or actions. To tell our professional readers that the practice of midwifery requires very often the most powerful resources of the mind, and the most dexterous application of the hand, would be to tell them what they all know; and appeals to the non-professional part of the community, we leave to those, whose arguments and statements will not bear the test of professional scrutiny. The growing sense of mankind, and, indeed, of womankind, is so completely in opposition to the proposition of once more placing midwifery in the hands of the weaker sex, that we should consider it an entire waste of time and space to occupy a single page with the discussion. Granting, as we freely do, to Sir Anthony Carlisle, the concession of good intentions and conscientious motives, we cannot but sincerely deplore the obliquity of judgment, which could lead a man, of otherwise cultivated and scientific mind, into a maze of errors, absurdities, and criminations, as unaccountable in their conception, as they are unjustifiable in their promulgation—as weak in argument, as they are mischievous in tendency—as baseless in their data, as false in their deductions—as discreditable to the author, as they are ungenerous and injurious to his obstetrical brethren.

*Carbonated Cheltenham Salts.*

Some of these salts have been sent to us, as prepared by Messrs. Beavan and Perrin, and we have found them to be one of the most agreeable aperients which we have ever employed. They are as pleasant to the palate as a common saline draught, and act mildly in the small dose of one or two tea-spoonfuls. They effervesce strongly by merely pouring water on them.

